# Precision medicine in rheumatoid arthritis: Evaluation of a new approach to predict clinical response to methotrexate in patients with early rheumatoid arthritis

**DOI:** 10.1371/journal.pone.0329440

**Published:** 2025-08-06

**Authors:** Alejandro Escudero, Mercedes Alperi, Ana Isabel Turrion, Rosario Lopez, Tatiana Yebra, Gema Cordero, Teresa Diez, Isabel Portero, Francisco J. Blanco

**Affiliations:** 1 Hospital Universitario Reina Sofia, Universidad de Cordoba, Cordoba, Spain; 2 IMIBIC Group GC05-Chronic systemic-inflammatory autoimmune diseases of the musculoskeletal system and connective tissue, Cordoba, Spain; 3 Hospital Universitario Central de Asturias, Oviedo, Spain; 4 Hospital Universitario de Salamanca, Salamanca, Spain; 5 Biohope Scientific Solutions for Human Health SL, Madrid, Spain; 6 Instituto de Investigación Biomédica de A Coruña (INIBIC), A Coruña, Spain; 7 Complejo Hospitalario Universitario de A Coruña (CHUAC), A Coruña, Spain; Nippon Medical School, JAPAN

## Abstract

**Background:**

International guidelines recommend methotrexate (MTX) as the first-choice treatment in rheumatoid arthritis (RA). Although most patients with recently diagnosed arthritis achieve low disease activity or remission with MTX, about 30–40% do not significantly decrease disease activity after 6-month treatment. Predicting response is essential for choosing the best therapeutic option during the window of opportunity.

**Objective:**

This study aimed to evaluate the performance of two new tests measuring the *in vitro* response to MTX in MTX-naive patients with RA and the association of the test results with clinical remission after 6-month treatment with MTX.

**Methods:**

This prospective 6-month study was conducted on 31 RA patients starting MTX treatment. Monocyte metabolic activity (Monocytes Test) and reactive oxygen species (ROS Test) in peripheral blood were measured *in vitro* before treatment, and response to MTX and remission was measured at 6 months. The area under the receiver operating characteristic curve (AUC) for predicting 6-month remission was calculated with 95% confidence intervals (CI) for each test.

**Results:**

Patients in remission (71%) and not in remission (29%) at 6 months showed no statistically significant clinical differences at baseline. They only differed in test results: ROS levels were higher in patients who achieved 6-month remission than in those who did not (p < 0.001), and monocyte levels were lower in patients who achieved remission than in those who did not (p < 0.05). Prediction accuracy was high, with AUC values of 0.919 (95% CI [0.813–1.025]) and 0.826 (95% CI [0.664–0.989]), respectively, for ROS and monocyte levels.

**Conclusions:**

In MTX-naive patients with RA, the pharmacological response to MTX can be adequately predicted *in vitro* by quantifying ROS production and total monocytes from peripheral blood mononuclear cells.

## Introduction

Methotrexate (MTX), a folic acid antagonist, is commonly used to treat many rheumatic and non-rheumatic diseases. MTX is considered the first-choice treatment in rheumatoid arthritis (RA) based on its efficacy, safety, and relatively low cost. It can be administered in monotherapy or combined with low-dose glucocorticoids, resulting in low disease activity or remission in almost half of patients with early RA [1]. Based on the recommendations of the American College of Rheumatology and the European Alliance of Association for Rheumatology (ACR/EULAR), MTX should be started early in recently diagnosed RA and undifferentiated arthritis to prevent joint destruction and disability [2–4]. Despite the development of several new targeted therapies, MTX remains the cornerstone of RA therapy due to its potent efficacy and tolerability, acting as an “anchor drug” [5–8].

Although some of the metabolic pathways on which MTX acts are known, its precise mechanism of action is still not fully understood. Different mechanisms have been proposed to explain the anti-inflammatory activity of MTX [9–12]. Adenosine signaling is probably the most widely accepted explanation for the MTX mechanism of action in RA given that MTX increases adenosine levels and, when adenosine interacts with its extracellular receptors, an intracellular cascade is activated, promoting an overall anti-inflammatory state [7,12,13]. In RA, it has been shown that macrophages display a pro-inflammatory polarization profile and that MTX-induced macrophage tolerance is mediated by upregulation of the NF-κB suppressor A20 [14]. Overall, MTX appears to act in RA as an anti-inflammatory agent with subtle immunomodulating properties and direct inhibitory effects on rapidly proliferating cells in the synovium.

The inter-individual heterogeneity in the clinical response to MTX is considerable in terms of efficacy and toxicity. The response rate can vary from 50 to 70% according to the ACR20 criteria [5,6]. Variability may be explained by factors related to the patient (age, gender, and comorbidity), the disease (duration and activity), and genetic polymorphisms (in MTX transport and metabolism, folate, and adenosine pathways) [7,10]. Nevertheless, the frequency of only moderate or no response after 3–4 months of MTX monotherapy in early RA is higher than 50% [15].

Predicting response to MTX is essential for choosing the optimum treatment for inflammatory control, especially in patients who will not respond. Many studies have assessed different predictors of response to MTX in RA patients, but few predictors have been validated, or their validation showed conflicting results [15–17]. Individual biomarkers, such as serum proteins [18–20], microRNA [21], and single nucleotide polymorphisms [22] have been studied as potential predictors, although results were inconsistent and have not yet been validated. A systematic review identified 100 different predictors, including seven related to patient characteristics, 22 linked to disease characteristics, 50 genetic predictors, and 23 other laboratory markers; none were related to the folate and adenosine pathways, and no metanalysis was deemed appropriate due to the heterogeneity of predictors, analysis types, definitions of clinical response, and time points of clinical response measurements in the included articles [23].

In addition to individual predictors, clinical prediction models using baseline information, combining clinical characteristics with genetic and other laboratory predictors, have also been developed to predict response to MTX in RA patients [23,24]. Machine learning approaches combining pharmacogenomic biomarkers and clinical data have also been explored [25,26]. However, all these models, based or not on artificial intelligence, have little impact on clinical practice.

Peripheral blood mononuclear cells (PBMCs) are key cells in the pathogenesis of RA. Monocytes, a type of PBMCs, have the ability to act as a bridge between the innate and adaptive immune systems and constitute an important target for MTX [27,28]. Their activation and migration to the synovial membrane is a central phenomenon in the appearance of the inflammatory manifestations of the disease. These cells are probably one of the critical targets for MTX. In this sense, the absolute number of monocytes in RA patients who are non-responders has been shown to be increased (compared to responders and healthy donors) after 6 months of MTX treatment [27,28]. However, the absolute number of monocytes may not be sensitive enough to detect inter-individual differences. We proposed an *in vitro* model to test the inhibitory effect of MTX on patients’ monocytes from blood samples as a pharmacodynamic biomarker of response. The overall cellular metabolic activity of the monocytes and their proliferation is measured after MTX exposure using the resazurin reduction assay, a known and validated redox-sensitive fluorochrome that turns color as a function of ‘metabolic intensity’ and the number of live cells present.

On the other hand, ROS is an innovative and largely unstudied measurement in this field. Contrary to the conventional view that elevated levels of ROS are involved in inflammation, it has been shown that reducing the ability to produce ROS promotes the activation of arthritogenic T cells leading to severe arthritis in murine models [29]. Therefore, increased redox activity should be expected in cultured PBMCs from MTX-responsive RA patients.

Based on these data, we proposed a proof-of-concept (PoC) study to evaluate the role of monocytes and ROS as pharmacodynamic biomarkers of response to MTX.

The current study aimed to evaluate the performance of two new tests based on changes in monocyte metabolic activity and ROS production to measure the *in vitro* response to MTX in patients with recent-onset RA and analyze the association of this response with clinical remission at 6 months.

## Materials and methods

In this longitudinal, observational, feasibility PoC study, early RA patients were consecutively included in three reference centers before starting MTX treatment and followed for six months. All patients signed a written informed consent form before being included in the study, which was initiated after obtaining the approval of the Ethical Committee.

Patients diagnosed with RA according to the 2010 American College of Rheumatology (ACR)-European League Against Rheumatism (EULAR) classification criteria for RA [2], older than 18 years, who had not received treatment with MTX or any other disease-modifying antirheumatic drug, and with a Disease Activity Score 28 (DAS28)≥2.6 were included. Patients with systemic infection by virus, bacteria, or fungi requiring antimicrobial treatment; human immunodeficiency virus, hepatitis, or other infectious agents preventing correct handling of clinical samples in the laboratory; treatment with biologics; low adherence; corticosteroids for ≥1 month and at a dose ≥15 mg of prednisone or equivalent on the day before study inclusion date; and severe systemic diseases or physiological situations that may affect the immune response, such as pregnancy or lactation, were excluded. The recruitment period began on May 29^th^, 2019 and concluded on March 2^nd^, 2020.

Clinical remission/response analyses were based on DAS28-erythrocyte sedimentation rate (ESR) according to recent EULAR recommendations and studies comparing the DAS28-ESR and DAS28-C-reactive protein (CRP) [30–32].

All patients received MTX weekly for 6 months, with dose adjustment as per standard clinical practice. Clinical remission/response was defined by a DAS28 value ≤2.6 at 6 months or a reduction ≥1.2 points in the score compared with the baseline value [30]. International guidelines recommend the use of MTX for six months before considering a change of treatment to a second line. Therefore, a 6-month follow-up period was considered [2–4].

Before starting MTX treatment, a 30mL blood sample was drawn to measure ROS and monocyte test performance in vitro.

Investigators and patients were blinded to the test results throughout the entire duration of the study.

### Isolation of peripheral blood

Blood samples were collected by venipuncture and stored in sodium heparin. PBMCs were isolated by density gradient centrifugation using a Ficoll medium. Then, PBMCs/cells were counted and frozen with an X-VIVO medium and a HyClone™ HyCryo cryopreservation medium.

### Cell activation and MTX treatment

After 3 weeks in liquid nitrogen, PBMCs were defrosted and brought to a concentration of 4M/mL with X-VIVO supplemented with human serum AB 1%. PBMCs were cultured in a p96 flat bottom plate. Then, 100 µL of distilled water were added to the wells of the p96 margins to prevent evaporation. After 24 hours, cells were activated with phytohemagglutinin (5 µg/ml) to induce lymphocyte proliferation among cultured PBMCs, and then exposed to MTX (5 µg/µL) for 48 hours. Three wells of untreated cells were left as positive controls, and three blanks contained only 100 µL of X-VIVO medium.

### Monocytes test

After 48 hours of culture exposure to MTX, the supernatant was removed from the wells and replaced by a fresh X-VIVO medium. The percentage of monocytes resulting from MTX effect was measured by means of the overall cellular metabolic activity of monocytes using the Resazurin solution (Presto Blue; Thermo Fisher Cat. A13261), which is used as an oxidation-reduction indicator in cell viability assays. Resazurin was diluted in X-VIVO medium at a ratio of 1:2 and then added with a pipette at a ratio of 1:10 relative to the final volume in each well. Plates were incubated for 2 hours (37ºC, 5% CO2) before fluorimetry reading. Monocyte fluorescence was measured using a Spark microplate reader (Tecan) in fluorimetry wavelength mode at 535/610nm emission/excitation (em/ex).

To generate a normalized representation of monocyte percentage, we measured the percentage of relative fluorescence units (% RFUs) with respect to the positive control (taken as 100%) and the blank control (taken as 0%).

### ROS test

To prepare the ROS stimulus, 1 µL of *Escherichia coli* stock bottle (Sigma MBD007, kept at −20°C, 10^8^ bacteria/mL) was added to 99 µL of buffer for an intermediate dilution. Another dilution was carried out in the same way to reach a final concentration of 500 bacteria/ml. Then, 10 µL of previously prepared *E. coli* were added. After 15 min, fluorescence was quantified using a Spark microplate reader (Tecan) in fluorimetry wavelength mode at 535/610 nm em/ex.

To generate a normalized representation of ROS production, we measured the % RFUs with respect to the positive control (taken as 100%) and the blank control (taken as 0%).

### Statistical analysis

Variables with nominal scales were described using absolute and relative frequencies. The Kolmogorov-Smirnov test was used to explore the normal distribution of continuous variables, and normally distributed variables were expressed as mean values and standard deviations.

Baseline comparisons between responders and non-responders at 6 months were performed using Pearson’s chi-square test, Fisher’s exact test, Student’s t-test, or Mann-Whitney U test for independent samples according to variable type.

ROS and monocyte levels of patients with and without response to MTX at 6 months were compared by using non-parametric tests (Mann-Whitney U).

Baseline values of monocyte metabolic activity and ROS values to predict response to MTX at 6 months of follow-up were assessed by receiver operating characteristic (ROC) curve analyses and the respective areas under the curve (AUCs).

## Results

### Baseline patient characteristics

The sample consisted of 31 RA patients who fulfilled the study selection criteria. The monocyte test was performed in 26 patients, and ROS test results were obtained in 25. In 20 of the 31 patients, both tests were carried out. For the remaining patients, performing both tests was impossible due to an insufficient cell number.

After 6 months of MTX treatment, the number of patients showing clinical remission was 17 in the ROS group (68%) and 18 in the monocyte group (69%). At baseline, no statistically significant differences were found between patients with and without remission according to age, gender, ESR, CRP, smoking habits, number of tender and swollen joints, DAS28-ESR value, rheumatoid factor (RF) and anti-citrullinated protein autoantibodies (ACPAs), the absolute number of monocytes as well as glucocorticoid use and dosage (Table 1). No patient presented extraarticular damage at baseline. Patients in the ROS group with clinical remission/response received a mean MTX dose at 6 months of 14.7 ± 4.8 mg/week, while the mean dose was 20 ± 5.9 mg/week in those without clinical remission/response. In the monocyte group, MTX doses were 15.3 ± 4.7 mg/week vs. 20 ± 5.9 mg/week, respectively. In the ROS group, four patients with clinical remission/response were also treated with hydroxychloroquine at 6 months, and only one in the group without clinical remission/response received additional leflunomide. In the monocyte group, only four patients with clinical remission/response also received hydroxychloroquine.

**Table 1:** Patient characteristics according to remission status at 6 months and test performed.

Variables	ROS test			Monocyte test		
	Remission status		*p*	Remission status		*p*
	Yes (n = 17)	No (n = 8)		Yes (n = 18)	No (n = 8)	
Age (years)	53.0 ± 20.1	56.9 ± 14.8	0.6	53.3 ± 19.4	57.9 ± 14.2	0.6
Female gender, n (%)	11 (64.7%)	8 (100%)	0.1	14 (78%)	8 (100%)	0.3
Smokers, n (%)	5 (30%)	1 (12.5%)	0.4	7 (39%)	1 (12.5%)	0.2
RF (IU/mL)	142.2 ± 198.2	117.8 ± 198.9	0.6	207.9 ± 235.9	180.4 ± 256.8	0.5
ACPAs (IU/mL)	151.4 ± 128.0	225.4 ± 202.8	0.4	156.8 ± 127.7	285.4 ± 205.1	0.1
ESR (mm/h)	30 ± 21	36 ± 28	0.7	34 ± 21	47 ± 34	0.5
CRP (IU/mL)	12.6 ± 17.2	15.1 ± 15.9	0.3	12.4 ± 16.6	22.0 ± 22.8	0.2
Tender joint count (>28)	9.1 ± 7.2	7.1 ± 5.2	0.6	7.7 ± 5.2	9.4 ± 7.9	0.6
Swollen joint count (>28)	5.5 ± 4.1	5.4 ± 3.9	0.9	4.9 ± 2.7	5.2 ± 3.9	0.9
Extra-articular involvement	0	0	NA	0	0	NA
DAS28-ESR score	5.31 ± 1.1	5.23 ± 1.4	0.9	5.37 ± 1.01	5.43 ± 1.5	0.8
Glucocorticoid use (%)	64.7%	62.5%	NA	66.0%	62.5%	NA
Glucocorticoids (dose, mg/day)	7.2 ± 4.8	6.8 ± 3.3	0.9	8.5 ± 6.7	10.8 ± 7.9	0.6
Monocyte percentage (%)	6.4 ± 1,7	6,65 ± 1.2	0.8	6.37 ± 2.1	6.61 ± 3.5	0.8
Total monocytes (10e3/mcl)	0.6 ± 0.4	0.55 ± 0.3	0.7	0.56 ± 0.4	0.56 ± 0.25	0.9

Values are expressed as mean±SD (standard deviation) unless otherwise noted.

RF = rheumatoid factor; ACPAs = anti-citrullinated peptide antibodies; ESR = erythrocyte sedimentation rate; CRP = C-reactive protein; ROS = reactive oxygen species; IU = international units; DAS28 = disease activity score; NA = not applicable.

### Test results

**Fig 1:**
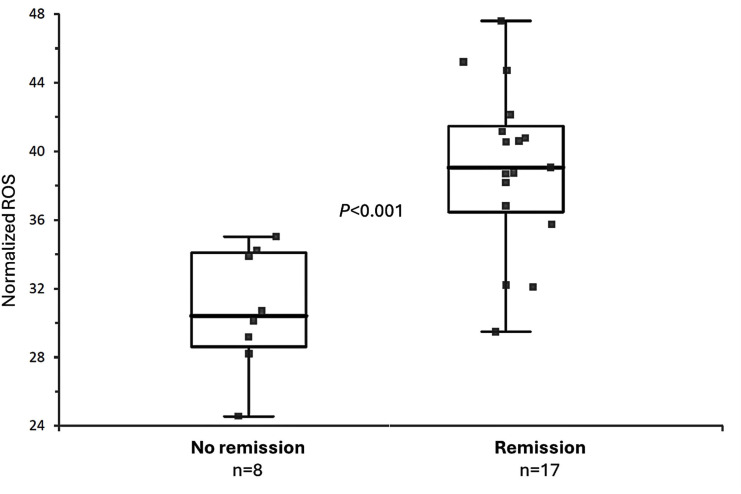
ROS production in patients with and without response to MTX after 6 months. ROS (fluorescence units) production of PBMCs. NO: naïve RA patients not in clinical remission after 6 months of methotrexate treatment (n = 8). YES: naïve RA patients in clinical remission after 6 months of methotrexate treatment (n = 17) (total n = 25).

**Fig 2:**
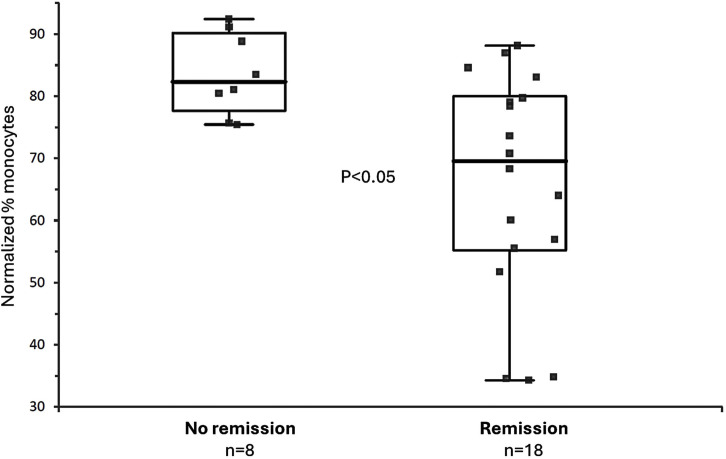
Monocyte percentage levels in patients with and without response to MTX after 6 months. Percentage of monocytes. NO: naive RA patients who do not show clinical remission after 6 months of methotrexate treatment (n = 8). YES: naive RA patients who do show clinical remission at 6 months of methotrexate treatment (n = 18) (total n = 26). Both ROS and monocyte tests displayed good accuracy in predicting response to MTX. ROC curves for ROS (Fig 3) and monocyte tests (Fig 4) showed AUC values of 0.919 (95% CI [0.813-1.025]) and 0.826 (95% CI [0.664-0.989]), respectively.

ROS production and monocyte percentage levels determined at baseline *in vitro* with the ROS test and with the monocytes test significantly differed between patients with and without response to MTX at 6 months. ROS levels were lower in patients without remission (30.8 ± 3.84%) than in those with clinical remission (37.92 ± 4.28%) (p = 0.0003) (Fig 1). The opposite occurred with monocyte levels, as they were lower in patients in remission (65.81 ± 18.1%) than in those not in remission (83.58 ± 6.62%) (p = 0.01) (Fig 2).

**Fig 3:**
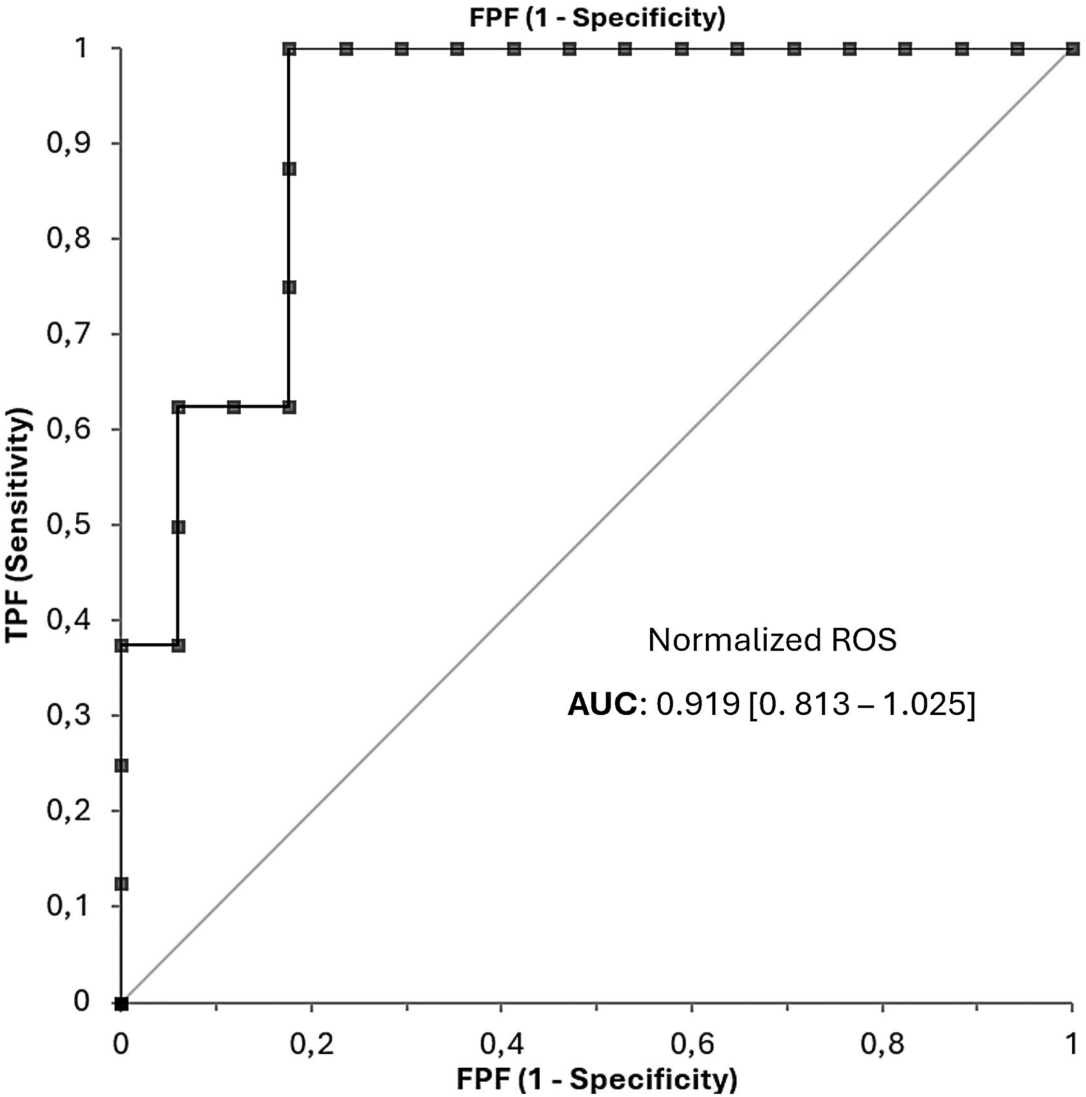
Predictive accuracy of the ROS test: ROC curves. ROC curve plot of the ROS test in patients with new-onset rheumatoid arthritis who did and did not show clinical remission in response to methotrexate.

**Fig 4:**
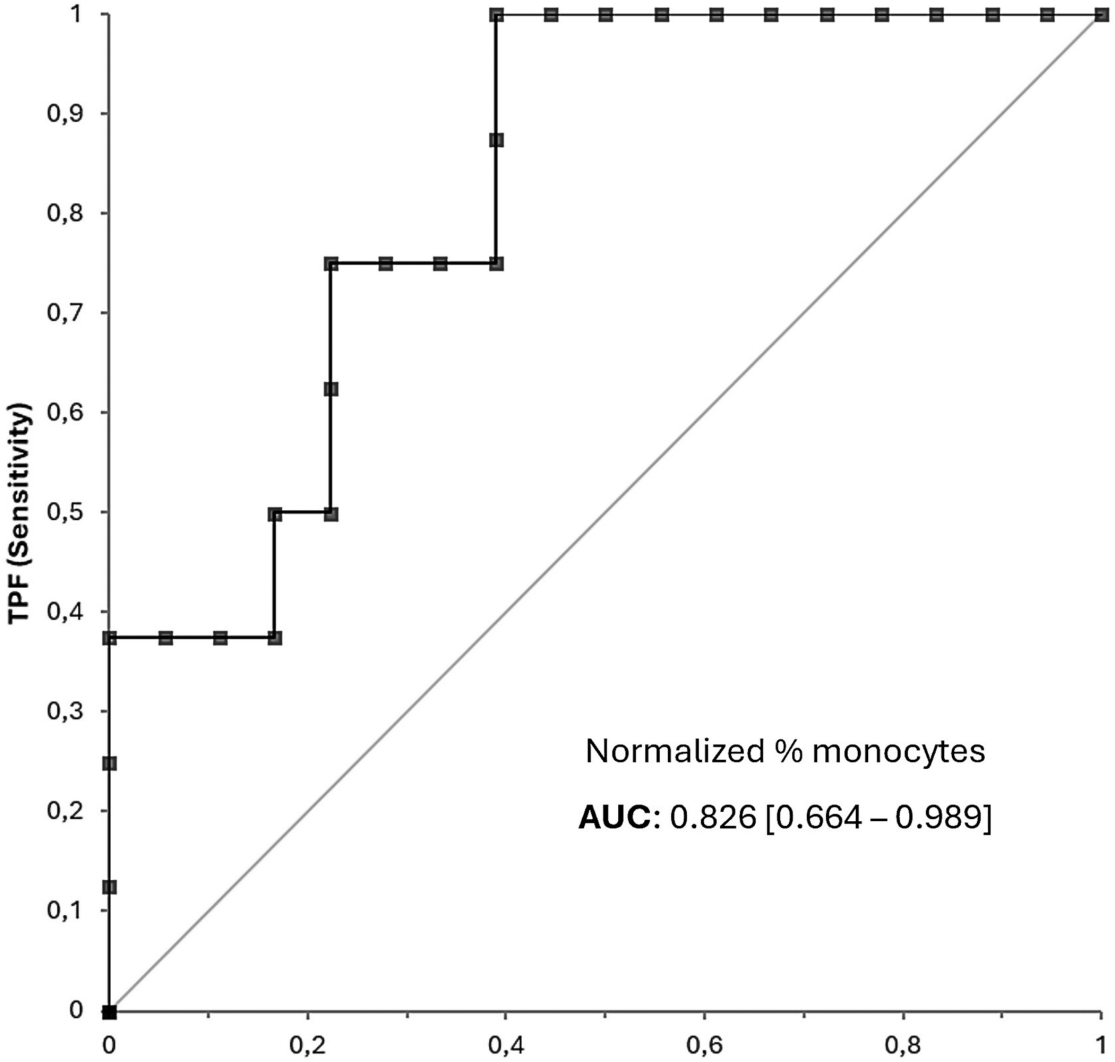
Predictive accuracy of the monocytes test: ROC curves. ROC curve plot of the Monocyte test in patients with new-onset rheumatoid arthritis who did and did not show clinical remission in response to methotrexate.

## Discussion

In this feasibility PoC study, two novel *in vitro* tests based on ROS and monocytes were evaluated in patients with early RA as predictors of the MTX response after 6 months of treatment. Baseline ROS and monocyte levels were significantly associated and accurately predicted clinical remission after 6 months of treatment with MTX.

Identifying clinical predictors of response to MTX might help limit MTX use to patients most likely to benefit from it and allow non-responders to be given more appropriate treatment more quickly. Currently no robust biomarker of response to MTX has been identified for routine use in clinical practice in RA patients. Based on the mechanism of action of MTX, our group hypothesized that the pre-treatment circulating monocyte number may predict clinical response to MTX [27], and that ROS production may be related to the pharmacological action of MTX [7,9]. The pre-treatment metabolic activity of circulating monocytes and the ROS levels after PBMC stimulation and *in vitro* exposure to MTX are thus potential biomarkers for response to MTX in RA patients. Our results suggested that both tests performed well for response prediction, with ROC AUC values of over 0.8.

The functions of B cells, including antigen presentation, cytokine secretion, and autoantibody production, are all related to RA pathogenesis. Monocytes play a central role in inflammation initiation and progression in RA due to the production of different mediators, such as interleukins and tumor necrosis factor, responsible for inflammation. Understanding the precise strategy targeting pro-inflammatory monocytes and macrophages could facilitate the identification of new biomarkers of disease activity or response to treatment in RA [28,33]. Some B cell subpopulations have been described as markers of response to MTX at 12 months [34].

Our group studied the role of monocytes, which can act as a bridge between the innate and adaptive immune systems and constitute an important target for MTX [27,28]. In a previous study, absolute monocyte number was measured by flow cytometry and was higher in non-responders than in responders to MTX. The peripheral blood monocyte count remained elevated throughout the whole 6-month study period. This prediction model has not been further validated [27]. We studied a cohort of 31 previously untreated RA patients and found that the baseline monocyte percentage and the total number of monocytes measured in the hemogram showed no significant differences between responders and non-responders. However, when these PBMCs were exposed *in vitro* to MTX, the monocyte percentage significantly increased in non-responders. The prediction model, which divided patients by monocyte count, yielded an ROC AUC of 89%.

Recently, Palmowski *et al*. studied a prediction model constructed via genome-wide gene expression analysis in CD4+ and CD14 + mononuclear cells with excellent predictions. If used in conjunction with previously identified clinical and laboratory (bio)markers, this model could help predict response to MTX at 3 months. However, validation in an independent cohort is required to support these findings [35].

As a prototype of chronic inflammatory autoimmune disease, RA has been linked to oxidative stress, a condition in which the ROS pool increases over time, ultimately implying impaired redox signaling. The exact mechanisms through which oxidative stress may contribute to inflammation initiation and perpetuation in RA, particularly during early stages, remain to be determined. Oxidative biomarkers have been used not only for complementary disease activity assessment but also for prognostic purposes [36].

The observed predictive value of monocyte percentage and ROS production for clinical response to MTX at 6 months in RA patients requires confirmation in large multicenter studies. Our study may have implications for future clinical practice if these results are confirmed in external validation studies.

A multivariate model combining both tests was not conducted due to the limited sample size. Nonetheless, future investigations involving larger cohorts should explore whether their combination, or other combinations with techniques that measure different cellular processes related to MTX mechanisms of action improve the overall predictive capacity of the models.

Despite the prospective design, which is a strength of this work, our study is not free of limitations. The sample size of this observational study was low. Additionally, there was a lack of a standardized treatment protocol, as this was not an interventional trial.

Approximately 20–30% of patients did not have enough cells to perform both tests mainly due to: a) difficulties to extract the minimum amount of blood necessary (30 ml), which decreases the cell yield; and b) deviations in the isolation and freezing of PBMCs at the different labs that can drive to a yield of less than 50% of viable cells at the time of thawing.

Our results should be validated in a larger cohort of RA patients. This study analyses possible predictive markers of response to MTX. We did not assess some previously identified markers, for example, the health assessment questionnaire (HAQ) was not used in the study and can be a significant remission predictor [15].

In this study, we observed a 6-month remission rate of 71% among patients with early-onset rheumatoid arthritis (RA) treated with methotrexate (MTX). This rate is slightly higher than those reported in comparable clinical studies, suggesting a potentially more favorable disease course in our cohort.

Several factors may account for this remission rate. Our sample included patients who showed a lower mean baseline disease activity (DAS28 near 5), less mean number of tender (7–9) and swollen (4–5) joint counts and a shorter disease duration than in other studies. Additionally, the disease duration at the time of recruitment was shorter. These characteristics are well-established predictors of better treatment outcomes and higher likelihood of remission.

The early referral of patients from primary care to specialized rheumatology services in our healthcare system may also play a critical role. Prompt initiation of MTX therapy in specialized settings likely contributes to improved disease control and remission rates.

The biomarker tests for response to MTX described in this work differ from previous attempts to predict response to MTX, and their predictability has been shown to be high.

Identifying predictive biomarkers can open possibilities for improving clinical practice outcomes by developing personalized treatment plans. These new diagnostic tests are important components of precision medicine approaches for RA treatment, personalizing the treatment regimen and ultimately improving disease outcomes.

In conclusion, in MTX-naive RA patients, the pharmacological response to MTX can be adequately predicted *in vitro* by quantifying ROS production and total monocytes from PBMCs. If validation studies confirm these results, our study may have implications for future clinical practice. Developing personalized treatment plans could improve disease outcomes.
